# Invasive maxillary sinus aspergillosis: A case report successfully treated with voriconazole and surgical debridement

**DOI:** 10.4317/jced.51571

**Published:** 2014-10-01

**Authors:** Beatriz Peral-Cagigal, Luis-Miguel Redondo-González, Alberto Verrier-Hernández

**Affiliations:** 1 Department of Oral and Maxillofacial Surgery. Río Hortega Hospital, Valladolid, Spain

## Abstract

Introduction: Invasive aspergillosis of the paranasal sinuses is a rare disease and often misdiagnosed; however, its incidence has seen substancial growth over the past 2 decades. Definitive diagnosis of these lesions is based on histological examination and fungal culture. Case Report: An 81-year-old woman with a history of pain in the left maxillary region is presented. The diagnosis was invasive maxillary aspergillosis in immunocompetent patient, which was successfully treated with voriconazole and surgical debridement. Possible clinical manifestations, diagnostic imaging techniques and treatment used are discussed. Since the introduction of voriconazole, there have been several reports of patients with invasive aspergillosis who responded to treatment with this new antifungal agent. Conclusions: We report the importance of early diagnosis and selection of an appropriate antifungal agent to achieve a successful treatment.

** Key words:**Invasive aspergillosis, voriconazole, fungal sinusitis, antifungal agent, open sinus surgery.

## Introduction

The term “Aspergillosis” refers to an illness due to allergy, airway or lung invasion, cutaneous infection, or extrapulmonary dissemination caused by species of *Aspergillus*. The most frequent site of human infection is the lung. Sinusitis is a common disorder affecting approximately 20% of the population at some time during their lives. Fungal sinusitis constitutes 6-9% of all the rhinosinusitis. *Aspergillus* is the most common fungus affecting paranasal sinuses (1). Worlwide, *A. fumigatus* is the most common species, followed by *A. flavus*. The maxillary sinus is the most common sinus to be affected.

Fungal rhinosinusitis is classified into an invasive and a non-invasive form, depending on invasion of the mucosal layer and destruction of the bone. The non-invasive forms are allergic sinusitis and aspergilloma; this infection leads to destruction of the sinus mucosa and bone atrophy. Invasive aspergillus infections can be either limited [chronic or indolent] or fulminant [acute], with a rapid malignant course advancing relentlessly to the destruction of the nasal cavity, the sinuses and the adjacent structures such as the orbit and the brain within a few days (2). The differential diagnosis of fulminant aspergillosis must include mucormycosis, pseudomona´s orofacial lesions or Wegener´s granulomatosis.

*Aspergillus* species are ubiquitous in nature, exposure and inhalation of their spores is a frequent event; however, the spores are sometimes introduced to the antrum via an oroantral communication that occurs at the time of a dental procedure such as a root canal perforation or a dental extraction. When the spores are inoculated into anaerobic sinuses they may become pathogenic (3). Tissue invasion is uncommon and occurs most frequently in the setting of immunosuppression associated with therapy for hematologic malignancies, hematopoietic cell transplantation, or solid organ transplantation. Neutropenia and glucocorticoid use are the most common predisposing factors. Recently, the disease has been increasingly reported in immunocompetent patients as well.

Histopathology, invasive aspergillosis is characterized by progression of the infection across tissue planes. One hallmark of infection is vascular invasion with subsequent infarction and tissue necrosis. The prognosis of invasive fungal sinusitis is potentially fatal, with an extremely high mortality rate, particularly in immunocompromised patients.

## Case Report

**Figure 1:**
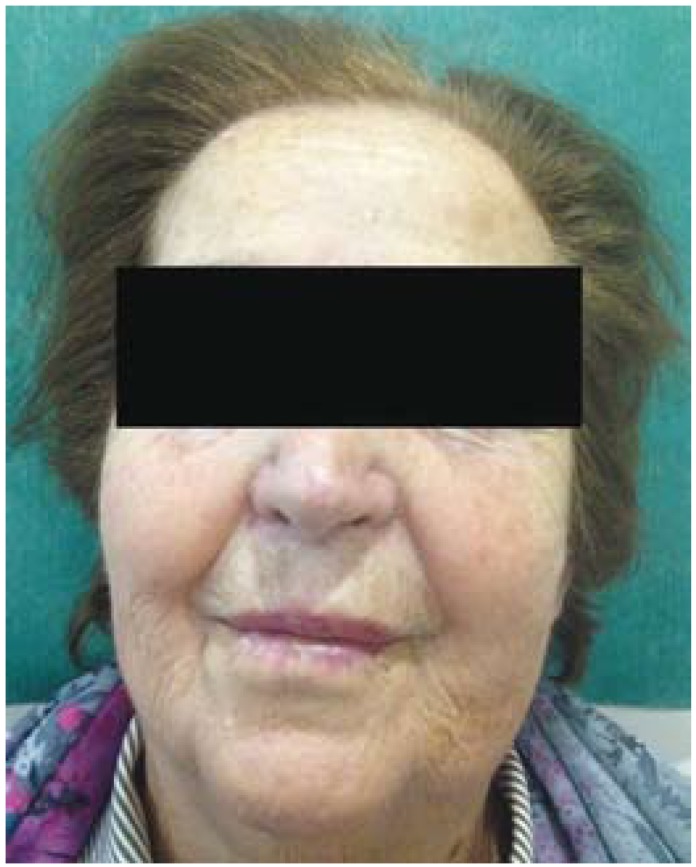
The patient presents a mild swelling in the left side of face.

An 81-year-old woman with a history of pain in the left maxillary region for 3-4 months was referred to our Department of Oral and Maxillofacial Surgery. She had a previous history of tooth extraction [maxillary left lateral incisor] 5 months before. There was no history suggestive of nasal obstruction, fever, diabetes or recurrent infections. The physical examination revealed a mild swelling affecting the left side of face (Fig. 1). The patient showed no evidence of immunosuppression after biologic investigations. A panoramic radiograph demonstrated opacification of the left maxillary sinus.

**Figure 2:**
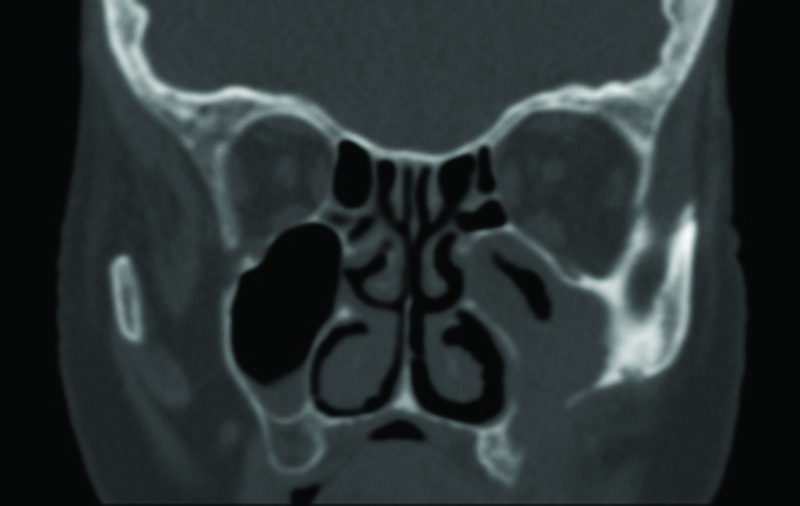
CT scan shows an opacification of left maxillary sinus with evidence of bone destruction of the anterolateral wall.

CT scan revealed a soft tissue density mass in the left maxillary sinus with bone erosion of the anterolateral wall, suggestive of a malignancy (Fig. 2). With this suspicion, we decided to perform a left Caldwell-Luc procedure under local anaesthesia to biopsy the mass and sent for histologic analysis. Histopathological examination showed abundant septate fungal hyphae with dichotomous branching suggestive of the Aspergillus species, with non-caseating granulomatous inflammation, eosinophils and giant cells.

**Figure 3:**
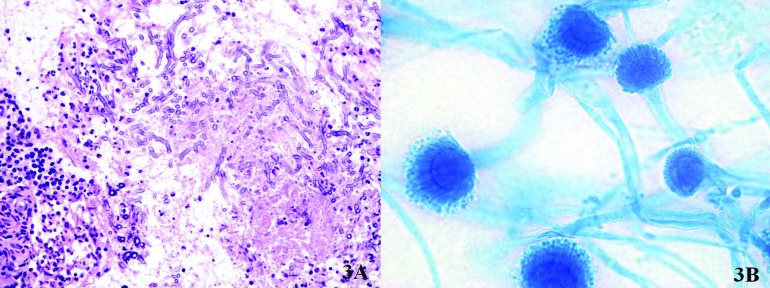
A.Histological examination (hematoxylin and eosin stain) shows abundant septate fungal hyphae with dichotomous branching suggestive of the *Aspergillus* spp., with non-caseating granulomatous inflammation, eosinophils and giant cells; B. Microscopic morphology of *A. fumigatus* (100x; lactophenol blue stain): hyphae are septate and the coniodophore is enlarged at the tip, forming a swollen vesicle.

With the diagnosis of fungal sinusitis, we decided to operate on the patient. A Caldwell-Luc procedure of the maxillary sinus was performed under general anaesthesia and both the granulation tissue and necrotic bone were removed, and the natural ostium of the maxillary sinus was widened. Macroscopically, bone destruction of the anterolateral and posterior walls of the maxillary sinus were seen. Histological examination showed the typical septate hyphae of *Aspergillus* with tissue invasion (Fig. 3), and the culture confirmed the species A. fumigatus (Fig. 3).

The medical treatment with intravenous voriconazole was started the day before surgery and continued for ten days [2 doses of 6 mg/kg on day 1, after 4 mg/kg twice daily], followed by 200 mg orally twice daily. After 4 months of oral administration of voriconazole, the patient remained well and CT scan showed no radiological evidence of disease, therefore we decided to interrupt the treatment. To this date the patient is asymptomatic and shows no clinical or radiographic evidence of recurrent disease.

## Discussion

Fungal infections of the paranasal sinuses need to be recognised in order to avoid significant mortality and morbility. Suspicion can arise in cases of purulent rhinosinusitis which do not respond to two or more courses of antibiotics, and on the basis of radiological features. Most invasive aspergillosis is caused by *A. fumigatus* [80-90%]; *A. flavus* [5-10%], *A. niger* [1-5%] and *A. terreus* [1%] are less common (4).* Aspergillus fumigatus* is the most common organism in immunocompetent patients too (5).

Predisposing factors that promote fungal infections in the sinuses include polyps and stagnant secretions besides other factors like neutropenia, inappropriate use of antibiotics, immunosuppressive drugs, corticosteroids, uncontrolled diabetes mellitus, human immunodeficiency virus infection, trauma, burns, and radiation therapy (6). Invasive aspergillosis is a major cause of death in immunosuppressed patients, particularly following hematopoietic cell transplantation.

In the paranasal sinuses, headache, nasal congestion, fever, and pain in the face and around the eye are common presenting features. Aspergillosis should be suspected in patients with refractory or recurrent sinusitis. Invasive aspergillosis originating from the nose and paranasal sinuses can cause an intra-orbital and intra-cranial growth mainly along the skull base and larger vessels. If the orbit becomes involved, additional symptoms may include blurred vision, gradual loss of vision, chemosis and proptosis. The infection can also extend locally into the vasculature and the brain, leading to cavernous sinus thrombosis and a variety of central nervous system manifestations (7). Intracranial and intraorbital extension decrease the survival rate and increase surgical morbidity.

Both magnetic resonance imaging [MRI] and CT scan can help to establish a diagnosis of invasive fungal sinusitis. Opacity of the sinus with or without destruction may be demonstrated in the invasive form. Although bone erosion and extrasinus extension are the classic CT findings highly suggestive of invasive fungal sinusitis, these are usually found late in the course of the disease; the most common early sign is severe unilateral nasal cavity mucosal and soft tissue edema (8). Bone involvement and erosion is more delineated on CT, while soft tissue extensions, vascular invasion and cavernous sinus involvement are more appreciated on MRI (1). Clinico-radiological findings can be misleading as the lesions are locally destructive and mimic a neoplasm.

Biopsy is necessary to establish the diagnosis. Hyphae are typical and specific for each fungus; *Mucor* presents large, broad non-septate hyphae with right-angle branching, and *Aspergillus* shows septate hyphae that branch at 45º angles. The histology should be specific as to whether there is mucosal involvement [invasive] or the mucosa is intact [non-invasive disease]. Fungal cultures on Sabouraud´s dextrose agar are needed to confirm the diagnosis.

Because of its rarity in immunocompetent patients, accurate recognition is critical in order to achieve optimal results. Delayed diagnosis and treatment of invasive maxillary sinus aspergillosis may lead to a poor therapeutic outcome.

Management of invasive aspergillosis therapy is still controversial and depends on several factors such as the nature of the disease, host immunity and degree of tissue invasion. Response to treatment depends on early diagnosis and initiation of antifungal therapy augmented by surgical debridement. Surgical debridement of abnormal tissue in the sinus is recommended for pharmacological therapy to reach the infected area. Surgery may improve the control of fungal disease and patient survival.

The Infectious Diseases Society of America [IDSA] released updated guidelines for the treatment of invasive aspergillosis in 2008. Voriconazole [broad-spectrum triazole] has now become the drug of choice for invasive aspergillosis (9). This is due to a better tolerance, increased efficacy [with a greater likelihood of a complete or partial response], improved survival [with a lower mortality rate], and significantly less toxicity when compared with amphotericin B (10). Only a few case reports have described the clinical course of invasive fungal sinusitis treated with voriconazole (10-13).

The recommended dosing regimen of voriconazole is 6 mg/kg IV every 12 hours on day one followed by 4 mg/kg IV twice daily, and after, 200 mg orally twice daily. The most frequent adverse events are visual disturbances, such as blurred vision, altered visual and colour perception, and photophobia. Other adverse events are skin reactions [rash, pruritus or photosensitivity], hepatotoxicity, visual hallucinations and confusion. All adverse effects described were transient and resolved when the drug was discontinued (11). In our case, the patient only had skin reactions like pruritus and dryness.

For decades, Amphotericin B deoxycholate has been the standard therapy for invasive aspergillosis although responses are suboptimal [less than 40 percent] in severely immuosuppressed patients. Amphotericin B is associated with multiple side effects [renal hepatic toxicity, anemia, fever and electrolyte abnormalities], which may decreased with the use of lipid formulations. In addition, amphotericin treatment requires intravenous administration and extended hospitalization. In patients who are intolerant of voriconazole, the IDSA suggests using a lipid formulation of amphotericin B at 5 mg/kg IV per day.

For the treatment of invasive aspergillosis of the sinuses, it is recommended to continue oral antifungal therapy for at least 4-6 months to prevent recurrence of the disease (13). The duration of therapy depends on several factors such as the location of the infection, the patient´s underlying disease and the need for further immunosuppression, and the response to therapy. For most patients, antifungal therapy will continue for months or even years in some cases. Regular post-operative follow-up is recommended in all the cases with CT scan and nasal endoscopy every 3-4 months. Early diagnosis of recurrent disease requires prolonged systemic antifungal chemotherapy.

The differential diagnosis of invasive aspergillosis includes benign and malignant neoplasms, syphilis, tuberculosis, sarcoidosis, Wegener´s granulomatosis, lymphoma, mucopyocele and allergic fungal sinusitis.

## Conclusions

Invasive maxillary sinus aspergillosis should be considered in the differential diagnosis of maxillary sinusitis that does not respond to standard conservative therapy with antibiotics even in immunocompetent patients.

Early diagnosis and therapeutic intervention, with selection of an appropriate antifungal agent and surgical debridement, is the key to successful treatment of invasive aspergillosis.

Voriconazole is recommended for the primary treatment of invasive aspergillosis in most patients; this is due to a better tolerance, increased efficacy, improved survival, and significantly less toxicity with fewer drug-related adverse events.
